# Pro-inflammatory TNFα and IL-1β differentially regulate the inflammatory phenotype of brain microvascular endothelial cells

**DOI:** 10.1186/s12974-015-0346-0

**Published:** 2015-07-08

**Authors:** Simon J. O’Carroll, Dan Ting Kho, Rachael Wiltshire, Vicky Nelson, Odunayo Rotimi, Rebecca Johnson, Catherine E. Angel, E. Scott Graham

**Affiliations:** Centre for Brain Research, School of Medical Sciences, Faculty of Medical and Health Sciences, University of Auckland, Auckland, New Zealand; Department of Anatomy, School of Medical Sciences, Faculty of Medical and Health Sciences, University of Auckland, Auckland, New Zealand; Department of Pharmacology and Clinical Pharmacology, School of Medical Sciences, Faculty of Medical and Health Sciences, University of Auckland, Auckland, New Zealand; School of Biological Sciences, Faculty of Science, University of Auckland, Auckland, New Zealand

**Keywords:** xCELLigence, Endothelial cells, CBA, Cytokine, IL-1β, TNFα, Human, ECIS

## Abstract

**Background:**

The vasculature of the brain is composed of endothelial cells, pericytes and astrocytic processes. The endothelial cells are the critical interface between the blood and the CNS parenchyma and are a critical component of the blood-brain barrier (BBB). These cells are innately programmed to respond to a myriad of inflammatory cytokines or other danger signals. IL-1β and TNFα are well recognised pro-inflammatory mediators, and here, we provide compelling evidence that they regulate the function and immune response profile of human cerebral microvascular endothelial cells (hCMVECs) differentially.

**Methods:**

We used xCELLigence biosensor technology, which revealed global differences in the endothelial response between IL-1β and TNFα. xCELLigence is a label-free impedance-based biosensor, which is ideal for acute or long-term comparison of drug effects on cell behaviour. In addition, flow cytometry and multiplex cytokine arrays were used to show differences in the inflammatory responses from the endothelial cells.

**Results:**

Extensive cytokine-secretion profiling and cell-surface immune phenotyping confirmed that the immune response of the hCMVEC to IL-1β was different to that of TNFα. Interestingly, of the 38 cytokines, chemokines and growth factors measured by cytometric bead array, the endothelial cells secreted only 13. Of importance was the observation that the majority of these cytokines were differentially regulated by either IL-1β or TNFα. Cell-surface expression of ICAM-1 and VCAM-1 were also differentially regulated by IL-1β or TNFα, where TNFα induced a substantially higher level of expression of both key leukocyte-adhesion molecules. A range of other cell-surface cellular and junctional adhesion molecules were basally expressed by the hCMVEC but were unaffected by IL-1β or TNFα.

**Conclusions:**

To our knowledge, this is the most comprehensive analysis of the immunological profile of brain endothelial cells and the first direct evidence that human brain endothelial cells are differentially regulated by these two key pro-inflammatory mediators.

**Electronic supplementary material:**

The online version of this article (doi:10.1186/s12974-015-0346-0) contains supplementary material, which is available to authorized users.

## Background

The vascular endothelium in the brain is the cellular interface between the blood and the central nervous system. These cells along with pericytes and vascular astrocytes form an essential physical barrier to immune cells, bacterial and viral infections (reviewed by [1, 2]). In addition, the nutrient homeostasis of the CNS is governed by the vascular endothelium, which is selective to or permeable to a range of small molecules and drugs [3]. Collectively, this is often referred to as the blood-brain barrier (BBB). Rather than being an impenetrable fortress, the BBB serves a range of critical functions including maintaining the brain’s immune privileged status (low frequency of leukocytes) and tightly regulating the transfer of nutrients and waste products [4, 5].

The main cellular component of the BBB is an uninterrupted layer of microvascular endothelial cells that are joined by tight-junctions (reviewed by [6]). These cells are highly polarised, with their basolateral aspect juxtaposed to a continuous protein-containing basement membrane (basal lamina). The presence of tight-junction complexes (e.g. claudin-5, occludin, ZO-1 (see [7]) seals adjacent endothelial cells and restricts paracellular transfer of large molecules and cells [6]. The apical aspect of the vascular endothelium is in direct contact with the blood and is therefore exposed to a range of insults including infections and systemic inflammatory responses.

It has become clear that the BBB is affected or changed in a wide variety of neurological diseases (e.g. multiple sclerosis [8, 9] and HIV infection [10, 11]), and in turn may modulate the disease process or clinical outcome [10, 12–14]. Although endothelial cells are not regarded as classical immune cells, extensive literature demonstrates that endothelial cells are inextricably involved in the inflammatory responses in many tissues. This is most likely very true in the human brain; however, most of what we believe about human brain endothelial (BBB) function has been inferred from observations using non-human or non-CNS-derived endothelial cells. This includes endothelial cells from other species [15–17] or from other tissues, such as the umbilical vein [18]. Understandably, the number of studies that have actually used human brain endothelial cells (either primary or immortalised) represents a minor proportion of the literature.

Inflammatory cytokines regulate the function of brain microvascular endothelial cells leading to changes in endothelial activation [19], inflammatory phenotype [19] and extravasation of immune cells [19–21]. Clinically, this likely contributes to the severity of disease where BBB dysfunction is evident. However, the temporal profile of these changes has not been studied in detail using human endothelial cells from the brain (or of brain origin). Here we compare the temporal response profiles of brain endothelial cells to several major pro-inflammatory regulators (IL-1β and TNFα) and reveal that the nature of the inflammatory response differs substantially. Initially, we used xCELLigence biosensor technology, which revealed that the global temporal response of the brain endothelial cells to IL-1β and TNFα was different. This observation was corroborated where expression or secretion of a number of key pro-inflammatory mediators were differentially regulated by IL-1β and TNFα. Using an extensive cytokine cytometric bead array (CBA) panel and flow-cytometry panel, we also advance our understanding of which cytokines, chemokines and adhesion molecules are expressed by brain endothelial cells. IL-1β or TNFα also induced changes in endothelial barrier strength measured as transendothelial electrical resistance (TEER) using electric cell substrate impedance-sensing (ECIS) technology. We observed complex differences in the changes in TEER induced by IL-1β and TNFα, where there were differences in the magnitude of the response and temporal nature of the response.

## Methods

### Cell culture of hCMVECs

The human cerebral microvascular endothelial cell (hCMVEC) line was purchased from ABMGood (USA see http://www.abmgood.com). Cells were maintained in growth media containing M199 media (Gibco) supplemented with 10 % FBS, 1 μg/mL hydrocortisone (Sigma), 3 μg/mL human FGF (Peprotech), 10 μg/mL human EGF (Peprotech), 10 μg/mL heparin (Sigma), 2 mM Glutamax (Gibco) and 80 μM butyryl cAMP (Sigma) referred to as M199 10 % growth media. The hCMVECs experiments were typically conducted using low-serum plating media containing M199 media, 2 % FBS, 110 nM hydrocortisone, 1 μM insulin and 80 μM butyryl cAMP (M199 2 % plating media).

### xCELLigence experiments

The xCELLigence real-time cell analyser (RTCA) measures cellular adhesion in real-time using custom E-plates, which are coated with high-density gold arrays for measuring electrical impedance (ACEA Biosciences, USA) [22, 23]. The xCELLigence biosensor measures cellular adhesion, which is converted to Cell Index (arbitrary units) by the xCELLigence software (version 1.2.1). Cells were seeded into E96 well plates at 54,000 cells per well in M199 2 % plating media and allowed to recover until the cells have attained a stable cell index. This was typically 24 h after seeding. Cytokine treatments are detailed in the corresponding figure legends.

### Immunocytochemistry for endothelial tight and adherens-junction proteins

For confocal imaging, the cells were grown to confluence on collagen-coated 8-chamber glass slides (BD Biosciences. Cat, no: 354108). Cells were fixed for immunocytochemical staining using 4 % PFA at room temperature for 10 min. Excess PFA was removed with several washes using PBS.

Primary antibody against VE-cadherin/CD144 (Santa Cruz Biotechnology Inc, sc-6458) was prepared in PBS containing 1 % donkey serum and used at 1:200 dilution. Primary antibody to zonula occludin-1 (ZO-1; Invitrogen, 339100) was prepared in PBS containing 1 % goat serum and used at 1:500 dilution. Primary antibodies were incubated at 4 °C overnight. Unbound antibodies were removed by washing in PBS containing 0.2 % triton-X100 (PBST). Fluorophore conjugated secondary detection was used using the species-specific anti-IgG Alexafluor 488 antibodies from Life Technologies (A-21206 and A11055). These secondaries were used at 1:400 and incubated with cells for 2 h at ambient. Again, unbound antibodies were removed with gentle agitation with PBST washes. Nuclei were counter stained using Hoescht (1:500 dilution in PBST), and cells were mounted using AF1 mountant. Confocal imaging was conducted using an Olympus FV100 microscope. Images were merged using ImageJ software.

### Stimulation of hCMVEC for cytokines production

The hCMVEC cultures were seeded into 24-well plates coated with 1 μg/cm^2^ collagen I (Gibco) at the density of 80,000 cells per well. Cells were grown to confluence in M199 10 % growth media. On the day of stimulation, the growth media was changed to M199 2 % plating media. In the first series of experiments, the stimulatory cytokines IL-1β and TNFα were each added to corresponding wells to the final concentration of 5 ng/mL. Control group received media only. 100 μl of conditioned media was collected at 4, 24 and 72 h post-treatment. In the second series of experiments, the cytokines were added across a range of concentrations (50 ng/mL to 50 pg/mL) for 24 h. The collected media were centrifuged at 400×*g* for 5 min at 4 °C to remove cellular debris. 80 μl of the clarified media was recovered and stored in several single-use aliquots for cytokine profiling. Media samples were stored at −20 °C.

### Cytokine measurements using cytometric bead array (CBA)

Soluble cytokines in the hCMVEC-conditioned media were measured simultaneously using multiplexed bead-based immunoassays, Cytometric Bead Array (CBA, BD Biosciences). The assay was conducted using 25 μl of sample and using a 10-point standard curve (ranging from 0 to 5000 pg/mL) was included for each cytokine measured (see Table 1 for list of cytokines). The samples were analysed using a BD Accuri C6 flow cytometer (BD Bioscience). FCAP Array software (BD version 3.1) was used to create the standard curves for each cytokine and convert the fluorescent MFI values into cytokine concentrations.

**Table 1:** Details of the cytokines measured in this study and whether they were secreted by the hCMVEC cultures

CBA flex set	Catalogue number	Secreted by hCMVECs^a^
Human Basic FGF Flex Set	558327	Yes
Human CD106 (VCAM-1) Flex Set	560427	Yes
Human CD54 (ICAM-1) Flex Set	560269	Yes
Human CD62E (E-Selectin) Flex Set	560419	No
Human CD62P (P-Selectin) Flex Set	560426	No
Human Soluble CD121a (IL-1 RI) Flex Set	560276	No
Human CD121b (IL-1 RII) Flex Set	560281	No
Human Eotaxin Flex Set	558329	No
Human CD178 (Fas Ligand) Flex Set	558330	No
Human Fractalkine Flex Set	560265	No
Human Granzyme B Flex Set	560304	No
Human GCSF Flex Set	558326	Yes
Human GMCSF	558335	Yes
Human IFN-γ Flex Set	558269	No
Human IL-1β Flex Set	558279	No
Human IL-2 Flex Set	558270	No
Human IL-4 Flex Set	558272	No
Human IL-6 Flex Set	558276	Yes
Human IL-7 Flex Set	558334	No
Human IL-8 Flex Set	558277	Yes
Human IL-9 Flex Set	558333	No
Human IL-10 Flex Set	558274	No
Human IL-13 Flex Set	558450	No
Human IL-17A Flex Set	560383	No
Human IL-17 F Flex Set	562151	No
Human IL-12/IL-23p40 Flex Set	560154	No
Human IL-21 Flex Set	560358	No
Human I-TAC Flex Set	560364	No
Human IP-10 Flex Set	558280	Yes
Human MCP-1 Flex Set	558287	Yes
Human MIG Flex Set	558286	No
Human MIP-1α Flex Set	558325	No
Human MIP-1β Flex Set	558288	No
Human RANTES Flex Set	558324	Yes
Human Soluble TNFRI Flex Set	560156	Yes
Human Soluble TNFRII Flex Set	560155	Yes
Human TNFα Flex Set	558273	No
Human VEGF Flex Set	558336	Yes

This panel comprises a list of chemokines, cytokines, soluble receptors and soluble adhesion molecules. The right-hand column summarises those cytokines that were secreted by the hCMVECs

^a^Under the conditions tested

### Cell-surface flow-cytometry analysis of hCMVEC

The hCMVEC cultures were seeded into T75 maintenance flasks and were grown to confluence in M199 10 % growth media. On the day of stimulation, the media was changed to M199 2 % plating media with 5 ng/mL IL-1β, 5 ng/ml TNFα or no stimulatory cytokine added for control group. After the designated treatment period (see figure legends) the endothelial cells were carefully harvested with EDTA-based Versene (Gibco). The use of trypsin was avoided as it cleaves many of the cell-surface epitopes. Cell suspensions were adjusted to the concentration of 1 × 10^6^ cells/ml with cold FACS buffer (PBS and 1 % FBS) in 100 μl per tube. Fluorochrome conjugated antibodies (see Table 2 for list of antibodies) were added to the cells at previously optimised dilutions and were incubated on ice for 10 min. Cells were washed twice with 1 ml of cold FACS buffer and were centrifuged at 400×*g* for 10 min. The supernatant was discarded, and cells were re-suspended in approximately 100 μl of FACS buffer. Flow cytometry was conducted using an Accuri C6 flow cytometer (BD Bioscience) calibrated with appropriate compensation controls. Each staining combination was incubated with 7AAD for live-dead cell determination. 7AAD-positive cells were ascribed to the dead gate (P2) and excluded from further analysis. 7AAD-negative cells represent the viable population and were ascribed as the live-gate (P1) (see Additional file 1: Figure S1 for further details). The specific staining of the flow antibodies (detailed in Table 1) was measured for the live-gate P1 only. The gating strategy for the flow-cytometry experiments is shown in Additional file 1: Figure S1.

**Table 2:** Details of the cell-surface adhesion molecules investigated in this study

Stain	Fluorochrome conjugate	Manufacturer	Catalogue number
CD102	PE	Biolegend	354503
CD105	Alexa Fluor 488	Biolegend	323209
CD106	PE	Biolegend	305806
CD138	APC	Biolegend	352307
CD141	APC	Biolegend	344106
CD144	PE	Biolegend	348506
CD146	Alexa Fluor 647	Biolegend	342005
CD147	Alexa Fluor 648	Biolegend	306209
CD150	PE	Biolegend	306307
CD304	PE	Biolegend	354503
CD31	Alexa Fluor 488	Biolegend	303110
CD321	FITC	Biolegend	353503
CD325	PE	Biolegend	350805
CD34	APC	Biolegend	343608
CD44	FITC	Biolegend	338803
CD49d	APC	Biolegend	304308
CD50	APC	Biolegend	330011
CD54	FITC	Biolegend	353108
CD62E	APC	Biolegend	336011
CD62L	APC	Biolegend	304809
CD62P	APC	Biolegend	304910
7AAD		BD Pharmingen	51-68981E

The table contains the marker CD name, fluorophore conjugate, manufacturer and catalogue number

### Measurement of TEER using ECIS technology

ECIS 96W20idf plates were coated with 10-mM L-cysteine to clean and modify the electrode surface so as to provide highly reproducible electrode capacitance. This was followed by coating the wells with 1 μg/cm^2^ collagen I. Then 50 μl of media was added to each well, and well stabilisation was carried out on the ECIS ZΘ to clean the electrodes prior to plating of cells. The hCMVEC cells were seeded at 20,000 cells/well and cultured for 48 h prior to treatment with cytokines as detailed in the corresponding figure legends. Multi frequency/time data was collected. Data were plotted at 4000 Hz, which is the optimal frequency to measure barrier formation and resistance [24].

## Results

### Analysis of hCMVEC response profile using xCELLigence biosensor technology

**Fig. 1:**
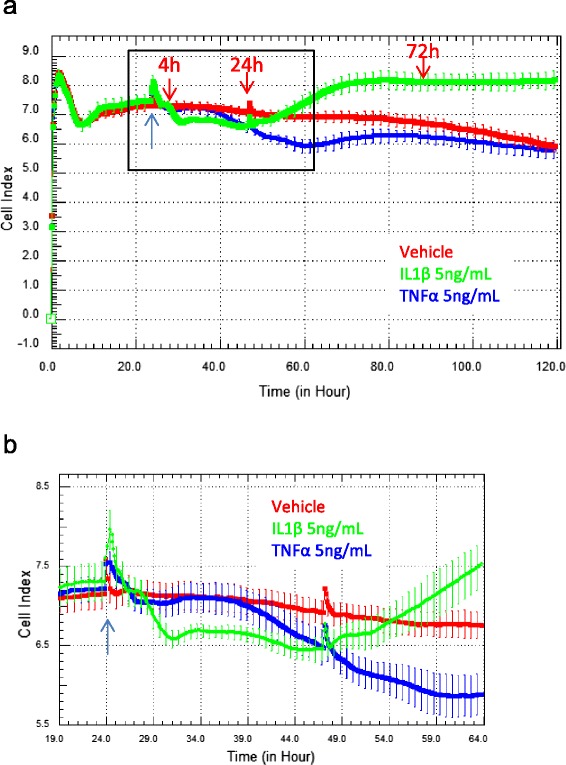
Analysis of pro-inflammatory IL-1β and TNFα activation of brain endothelial hCMVECs on cellular adhesion measured using xCELLigence Biosensor technology. **a** The net endothelial adhesion (cell index) was measured using xCELLigence across a 210-h period. Endothelial cells were treated with IL-1β (5 ng/mL; *green curve*) and TNFα (5 ng/mL; *blue curve*) 24 h after seeding. Cytokine addition is indicated by the *blue arrow*, whereas the *red arrows* highlight the time points when conditioned media was collected (from parallel plates). The control (vehicle cells) is indicated by the *red curve*. **b** The *curves* represent the mean of four individual well ± SD of a representative experiment

The real-time xCELLigence biosensor was used to ascertain the temporal response profile of the hCMVECs to IL-1β and TNFα (Fig. 1). The biosensor measures the net adhesion of living cells and is therefore ideal to reveal (a) if the cells respond and (b) when the cells respond to cytokine treatment. Figure 1a shows the long-term changes in endothelial adhesion induced by IL-1β and TNFα (both 5 ng/mL). Importantly, the profile suggests that the endothelial response to IL-1β and TNFα is not the same. IL-1β induces a sustained increase in endothelial adhesion (green curve) beginning approximately 24 h after activation, whereas with TNFα, the net endothelial adhesion is reduced for a period of ~100 following TNFα addition. Figure 1b focuses on the initial 24-h period after activation which shows when the cytokines were added (blue arrow) and that there is only a small change in net endothelial adhesion during this period, which is more obvious for the IL-1β treatment. In addition to the marked difference in net adhesion between the cytokine treatments, the long-term temporal profile indicates that neither treatment induces cytotoxicity. This is in contrast to the previous observation where we have observed IL-1β- and TNFα-induced compromise of astrocytes using xCELLigence technology [23]. Overall, the changes in endothelial adhesion induced by IL-1β are very different to the response to TNFα. This provides evidence that the endothelial response to these two key pro-inflammatory regulators is different.

### Characterisation of cytokine and chemokine secretion under basal and activated conditions

Multiplex cytokine analysis provides a powerful tool to assess complex secretory profiles of human immune cells [23, 25, 26]. This was employed to assess the basal secretory output of the hCMVECs and measure any changes following treatment with TNFα and IL-1β over a time course of 3 days. This period is highlighted by the red arrows in Fig. 1a.

**Fig. 2:**
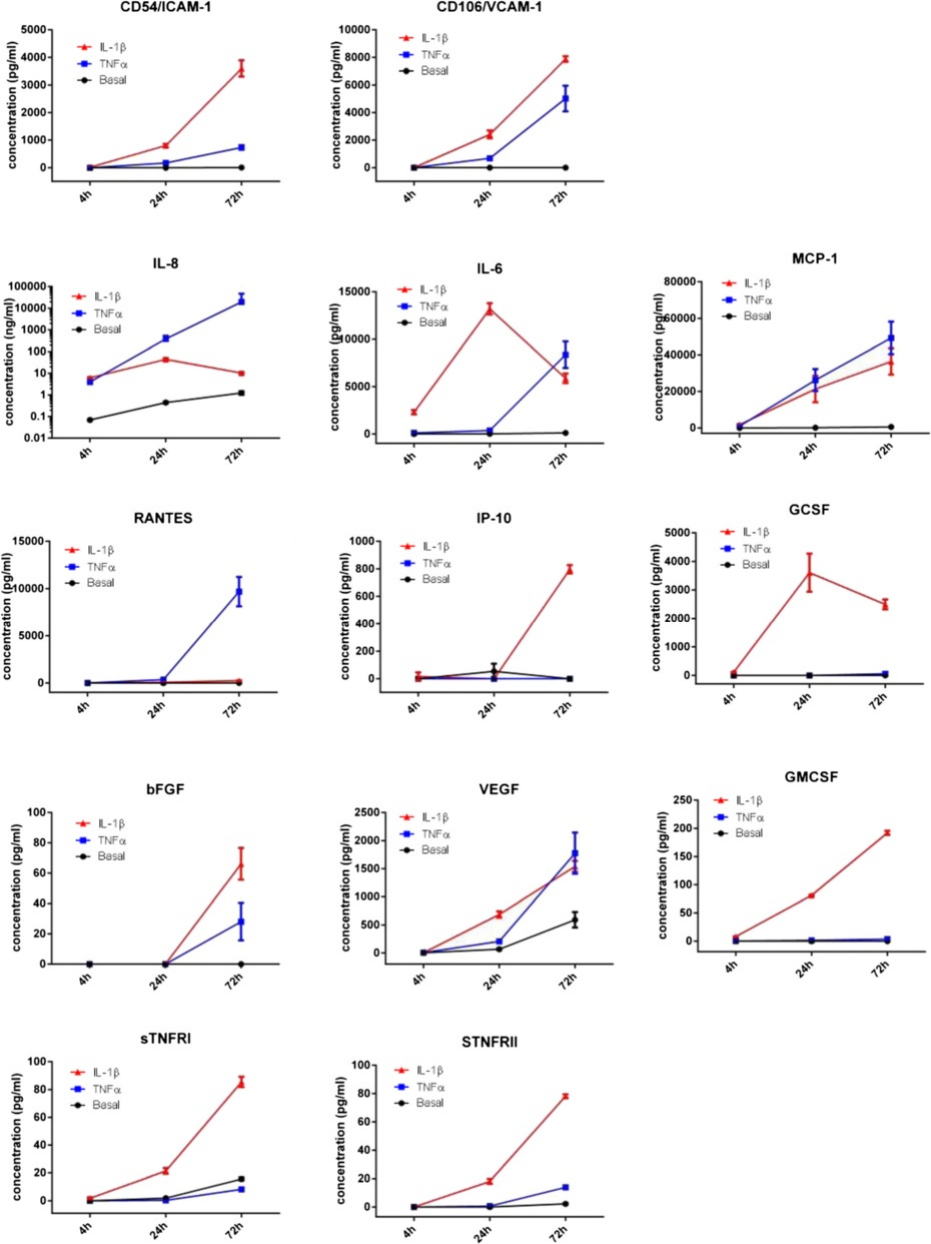
Characterisation of the cytokine-secretion profiles of hCMVEC cultures following activation by IL-1β and TNFα. The concentration of each secreted cytokine/chemokine was measured in conditioned media using multiplex CBA. Shown are the temporal secretion profiles (4 to 72 h) of the 13 cytokines detected out of a larger screening panel of 38 factors (see Table 1 for full panel of cytokines). Most were negligible or undetectable under basal conditions with secretion increasing following treatment with either IL-1β (5 ng/mL) or TNFα (5 ng/mL) treatment. Data show the mean ± SEM (*n* = 3)

In total, we measured the concentration of 38 different secreted cytokines, chemokines, growth factors and liberated soluble receptors in endothelial-conditioned media (see Table 1 for complete list of factors). The media were collected from brain endothelial cells maintained under basal (no activation) conditions or following activation with TNFα, IL-1β (Fig. 2) or PMA (see Additional file 2: Figure S2) across a time course of 72 h. In total, 13 of the 38 factors (see Table 1) were detected in the endothelial-conditioned media at above 1 pg /mL (lower limit of sensitivity). These were soluble ICAM-1 (sICAM-1/sCD54), sVCAM-1(sCD106), IL-6, IL-8 (CXCL8), MCP-1 (CCL2), RANTES (CCL5), IP-10 (CXCL10), VEGF, bFGF, GCSF, GMCSF, sTNFRI and sTNFRII. The concentration of most of these under basal conditions was low or undetectable (<1 pg/mL). This profile is consistent with the endothelial cells having a non-activated/non-inflamed phenotype under basal culture conditions. The secretion of each of the 13 cytokines was greatly increased following inflammatory stimulation with TNFα or IL-1β (5 ng/mL). Interestingly, specific factors were preferentially induced by IL-1β (red curves), which included sICAM-1, IL-6, sTNFRI, sTNFRII, GCSF, GMCSF and the chemokine IP-10. In contrast, RANTES and IL-8 were preferentially induced by TNFα. The endothelial cells were clearly responsive to both TNFα and IL-1β; however, the secretory immune response to each was considerably different. Interestingly, we measured the presence of the soluble versions of ICAM-1, VCAM-1 and the receptors for TNFα and IL-1β. As expected, soluble ICAM-1 and VCAM-1 were undetectable in the endothelial-conditioned media during basal culture conditions. However, both were induced and liberated following IL-1β treatment, which resulted in a greater concentration of soluble adhesion molecules than TNFα. Soluble TNF receptors (sTNFRI and sTNFRII) were also detected in the endothelial-conditioned media; however, to our surprise, the concentration of both sTNFRI and sTNFRII was increased to a greater extent by IL-1β than by TNFα treatment. In contrast, we did not detect the soluble forms of the IL-1 receptor (sIL-1R1 and sIL-1R2) under any conditions.

The phorbol ester PMA, was also used to potentially activate a wider signaling network than that induced by TNFα or IL-1β. PMA increased secretion of most of those regulated by TNFα and IL-1β. The amount secreted was generally lower in comparison to the cytokine-induced responses. That said, this clearly shows that activation of the PKC-signalling network results in a pro-inflammatory response in brain endothelial cells. There were, however, no additional cytokines present in our panel of 38 (see Table 1) specifically regulated by PMA.

It is worth noting that under these conditions, the hCMVEC did not secrete the MIP1α or MIP1β, which are potent pro-inflammatory chemokines nor did we detect secretion of the anti-inflammatory cytokines IL-4 or IL-10 from the endothelial cells under any conditions. The temporal profile of secretion, although expensive to conduct, reveals that IL-6 and GCSF levels at 72 h post IL-1β activation are lower than the levels observed earlier at 24 h. This is an interesting observation which suggests that the endothelial cells have the capacity to assimilate or degrade some of the cytokines they secrete. This is also an important observation in terms of future experiments, as the timing of media collection has to be carefully considered in this regard to maximise sensitivity of the response, but avoiding this later period, where additional mechanism are at play.

**Fig. 3:**
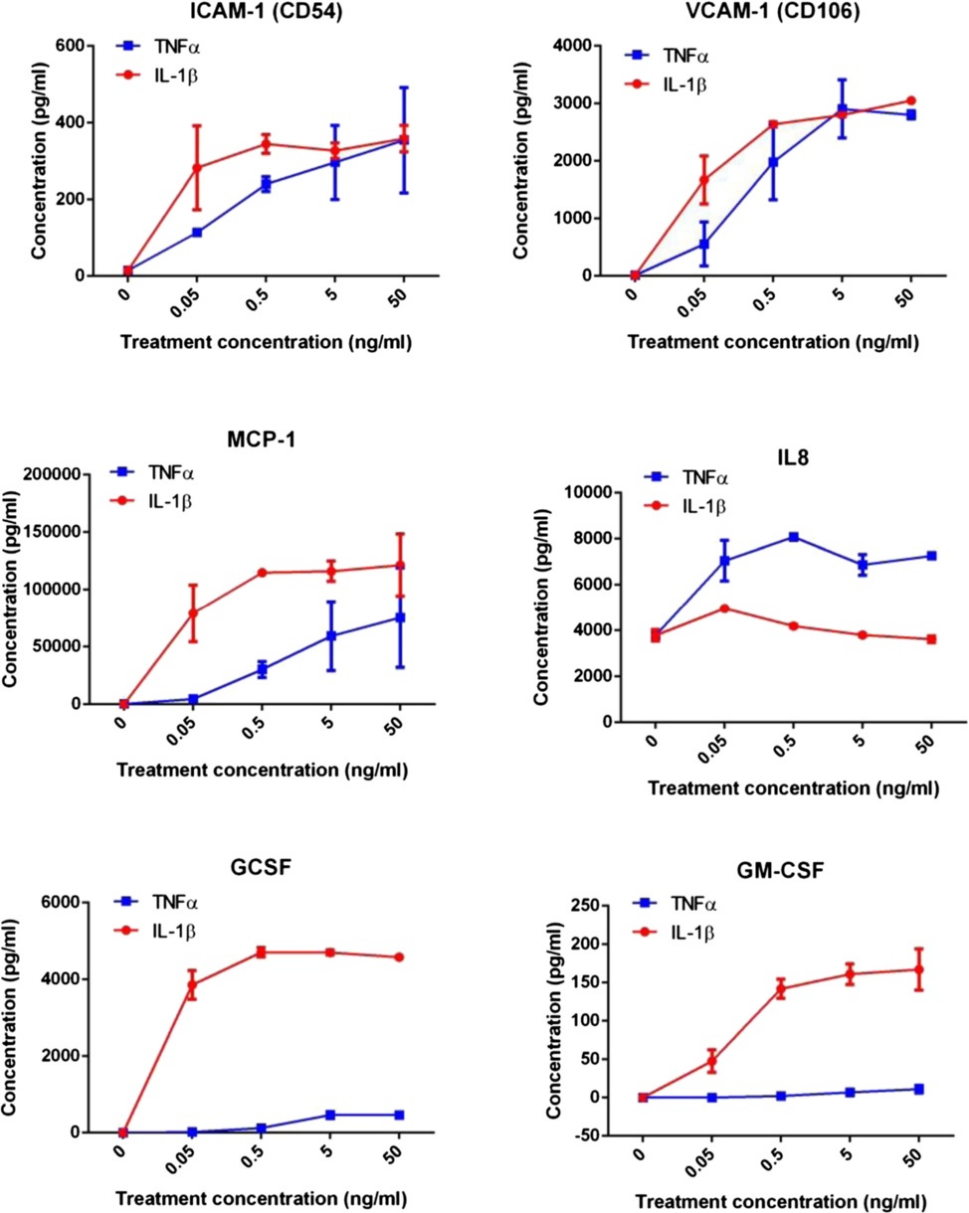
IL-1β and TNFα differentially regulate endothelial cytokine secretion. IL-1β and TNFα were added to the endothelial cultures across a concentration range of 50 pg/mL to 50 ng/mL, and secretions were measured by CBA after 24 h. The TNFα responses are shown in *blue* whereas the IL-1β responses are in *red*. Data show the mean ± SEM (*n* = 3)

As the data in Fig. 2 had highlighted pronounced differences in responsiveness to TNFα and IL1β, the cytokine-secretion study was extended to include a wider range of concentrations for TNFα and IL1β (50 pg/mL to 50 ng/mL; Fig. 3). Panels of cytokines were selected that were elevated at 24 h for this comparison. It is evident from Fig. 3 that IL-1β is considerably more potent than TNFα in terms of inducing secretion/release of sICAM, sVCAM, MCP-1, GCSF and GMCSF, whereas TNFα is the more potent inducer of the chemokine IL-8. In these data and also in the profiling conducted in Fig. 2, we would argue that there is only a minimal effect of TNFα on the induction of GCSF and GMCSF, in comparison to the effect of IL-1β. This further supports the observation that TNFα and IL1β are causing differential inflammatory responses in the hCMVECs.

### Cell-surface immunophenotyping of human brain microvascular endothelial cells

**Fig. 4:**
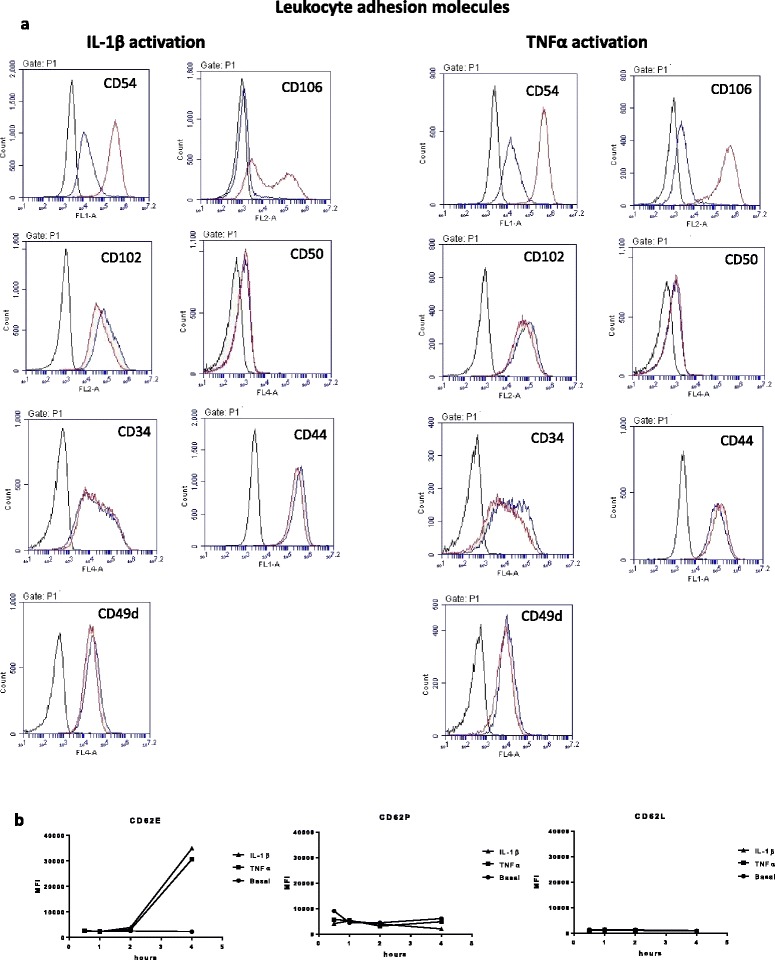
Analysis of IL-1β and TNFα regulation of key adhesion molecules involved in leukocyte adhesion. **a** The flow plots show the cell-surface expression levels of adhesion molecules 24 h after cytokine treatment (5 ng/mL). Of this panel, IL-1β and TNFα treatments appear to substantially increase CD54 (ICAM-1) and CD106 (VCAM-1) expression only. Note also the bimodal distribution for CD106 following IL-1β activation. The *black line* denotes the autofluorescence of the cells. The *blue curve* represents the basal level of expression, whereas the *red curve* is the expression level following cytokine treatment. **b** Time course of selectin (CD62E, P and L) expression following cytokine activation of the hCMVECs. These data are from a single experiment, which is representative of at least three independent experiments

**Fig. 5:**
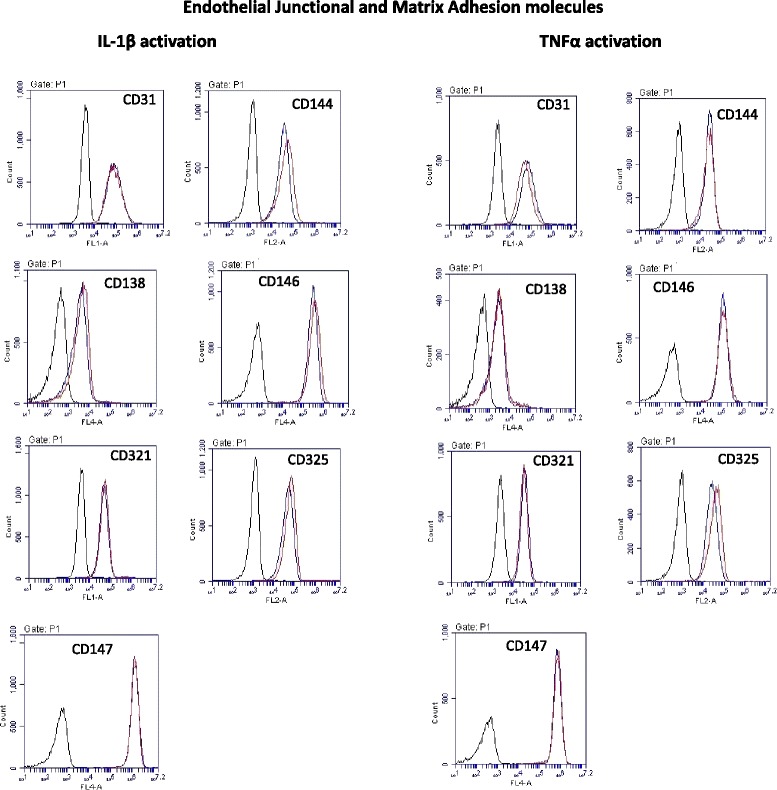
Analysis of IL-1β and TNFα regulation of adhesion molecules involved in basolateral matrix interactions. The flow plots show the cell-surface expression levels of adhesion molecules 24 h after cytokine treatment (5 ng/mL), with the specific adhesion molecule denoted in the *top* of the panel. The *black line* denotes the autofluorescence of the cells. The *blue curve* represents the basal level of expression, whereas the *red curve* is the expression level following cytokine treatment. These data are from a single experiment, which is representative of at least three independent experiments. Additional adhesion molecules were also investigated, and these are shown in Additional file 3: Figure S3

**Fig. 6:**
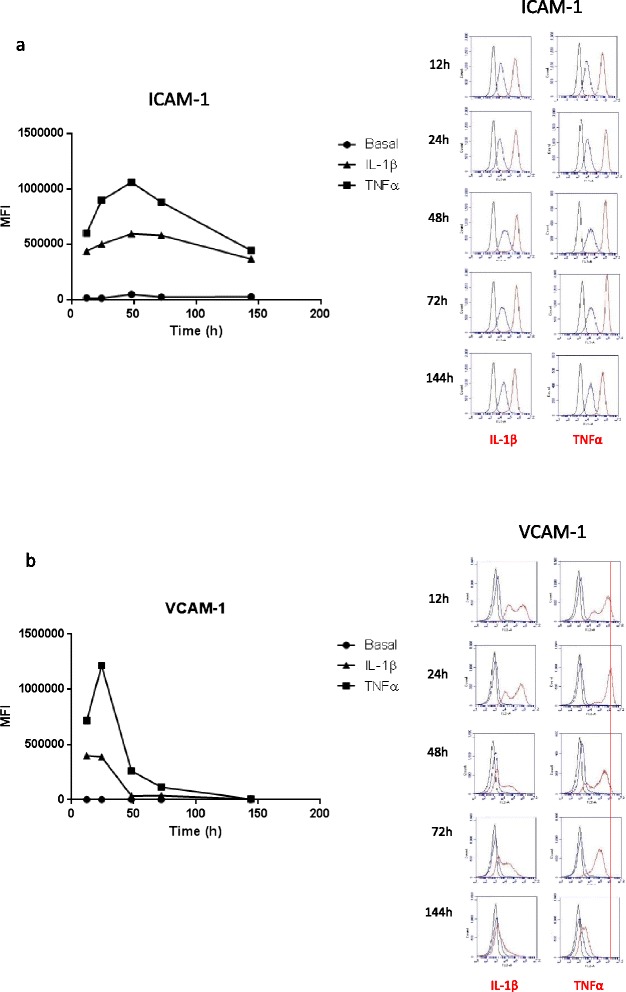
Time course of ICAM-1 and VCAM expression following IL-1β and TNFα activation reveals a time- and cytokine-dependent profile of expression. Cell-surface expression of (**a**) CD54 (ICAM-1) and (**b**) CD106 (VCAM-1) over a 6-day period under basal condition, following endothelial activation with IL-1β (5 ng/mL) or TNFα (5 ng/mL). Expression levels are represented as MFI (mean fluorescent intensity). The *right-hand panels* show the individual flow plots to highlight the bimodal expression profile present for VCAM-1. Data are representative of three independent experiments

The CBA cytokine profile strongly suggested that the endothelial response following IL-1β activation created a different inflammatory milieu in comparison to TNFα (see Fig. 2). It was notable that IL-1β induced the liberation of more soluble ICAM-1 and VCAM-1 in comparison to TNFα. We therefore hypothesised that these differences would also be reflected in the cell-surface expression level of these and other cell-adhesion molecules (CAMs). We therefore undertook an extensive analysis of the cell-surface immunological phenotype of the hCMVEC cells. This comprehensive analysis included leukocyte endothelial CAMs (see Fig. 4), adherens-junction molecules (see Fig. 4), endothelial-matrix adhesion molecules and various others (see Figs. 5 and 6 and Additional file 3: Figure S3).

As shown in Figs. 4 and 5 and Additional file 3: Figure S3, these brain microvascular endothelial cells basally express a range of adhesion molecules commonly expressed by endothelial cells from a variety of tissues (including CD31, CD34, CD138, CD141, CD144 and CD146). It should be noted that very few of these markers are truly endothelial-specific, which includes the commonly used CD31 and CD34 molecules (also expressed by some leukocytes). In addition, we also investigated expression and localisation of VE-cadherin and ZO-1 in the endothelial cells using immunocytochemistry (see Additional file 4: Figure S4). This confirmed the high level of expression of these two proteins as expected of brain-derived endothelial cells, which have critical roles in controlling endothelial barrier integrity. Of the entire panel of CAMs investigated in this study, it was E-selectin (CD62E), ICAM-1 (CD54) and VCAM-1 (CD106) that were consistently affected by TNFα and IL-1β. Importantly, basal expression of these was very low, which is consistent with a non-activated status. All of the selectin species were measured. However, only CD62E was induced by activation. Basal levels of P-selectin were very low, and L-selectin (as expected) was not expressed. Induction of CD62E was observed approximately 2–4 h after cytokine activation (see Fig. 3). Both ICAM-1 and VCAM-1 expression were substantially increased following activation by TNFα and IL-1β. Such activation-induced surface expression of these key leukocyte-attachment molecules is fully consistent with their role in leukocyte recruitment across the BBB. The data shown in Figs. 4 and 5 (except for the selectins), and Additional file 3: Figure S3 shows the expression of the cell-surface adhesion molecules 24 h after treatment. This was also conducted 72 h after treatment (data not shown), and only ICAM-1 and VCAM-1 showed substantial changes (summarized in Table 3).

**Table 3:** Expression of cell-surface adhesion molecules by brain endothelial cells

Molecules	Other names	Supporting literature	Expressed by the hCMVECs
CD102	ICAM2	Flow-cytometry analysis using hCMEC/D3 cells [34]	Yes
CD105	Endoglin	Flow cytometry using BMVEC [35]	Yes
CD106	VCAM1	RT-PCR using BMVEC [35]	Yes; highly inducible
CD138	Syndecan 1	Rat brain and rat brain-derived endothelial cells [36]	Yes
CD141	Thrombomodulin	IHC using human brain tissue (not expressed) [37]	Yes
		Regional distribution in human brain [38]	
CD144	VE-cadherin	ICC using hCMEC/D3) [34], ICC using THBMEC [39]	Yes
		IHC using human brain tissue [40]	
CD146	MCAM; S-endo-1; MUC-18	Flow cytometry using BMVEC [35]	Yes
CD147	BSG		Yes
CD150	SLAMF1		No
CD304	Neuropilin-1	Functional assay using hCMEC/D3, and IHC-rat brain [41]	Yes
CD31	PECAM1	Flow cytometry using hCMEC/D3 [34]	Yes
		Flow cytometry and RT-PCR using BMVEC [35]	
		Flow cytometry and ICC using HBMEC [42]	
CD321	JAM1	ICC using hCMEC/D3[34], ICC using THBMEC [39]	Yes
		IHC on human brain microvessels [40]	
CD325	Neural cadherin (NCAD)		Yes
CD34	HPCA-1	Flow cytometry using BMVEC [35]	Yes
CD44	H-CAM, Pgp-1	Flow cytometry using hCMEC/D3 [34]	Yes
CD49d	Alpha 4 integrin	THBMEC [39]	Yes
CD50	ICAM3	Expressed by leukocytes but not detected on endothelial cells in human brain by IHC [43]	Yes; low
CD54	ICAM1	Flow cytometry using hCMEC/D3 [34]	Yes; highly inducible
CD62E	E-selectin	ELISA detection using HCEC [44]	Yes; highly inducible
CD62L	L-selectin	Not expressed	No
CD62P	P-selectin	IHC using human brain [45];	Yes; very low
		IHC using human infarcted brain [46]	

hCMEC/D3 are immortalised human brain endothelial cell lines from adult females with epilepsy (not commercially available). BMVEC are human brain endothelial cells from foetal brain tissue. THBMEC are immortalised adult transfected human brain microvascular endothelial cells. HCEC are primary adult human cerebromicrovascular endothelial cells. HBMVE are primary human brain microvascular endothelia. HBMEC are human brain-derived microvascular endothelial cell (primary cells)

The better known generic names for each adhesion molecule are detailed alongside the literature demonstrating expression or function of the given adhesion molecule in endothelial cells or in brain tissue. The final column summarises whether the adhesion molecule was expressed by the hCMVECs used in this study

*ICC* immunocytochemistry, *IHC* immunohistochemistry

There was a pronounced “bimodal” change in VCAM-1 expression following 24-h IL-1β treatment, but not as evident with TNFα (Fig. 4). It is possible that the left peak (lower expression) is related to the sVCAM-1 liberation in a population of cells as measured during the cytokine profiling. It was therefore decided to conduct a longer time course for these key leukocyte-adhesion molecules to ascertain their profile more fully. Figure 6 reveals the temporal profile of (a) ICAM-1 and (b) VCAM-1 following cytokine activation of the hCMVECs over a 144-h time course. Of particular interest is that TNFα induces a markedly higher level of both ICAM-1 and VCAM-1 expression in comparison to IL-1β. This time course reveals that both ICAM-1 and VCAM-1 expression occurs quickly and, in the case of ICAM-1, is sustained at high levels for a number of days. In contrast, VCAM-1 expression is shorter lived and peaks around 24 h (highlighted by the red line). The temporal breakdown (right-hand plots) reveals that the increased VCAM-1 expression is bimodal for both IL-1β and TNFα treatments (Fig. 6).

**Fig. 7:**
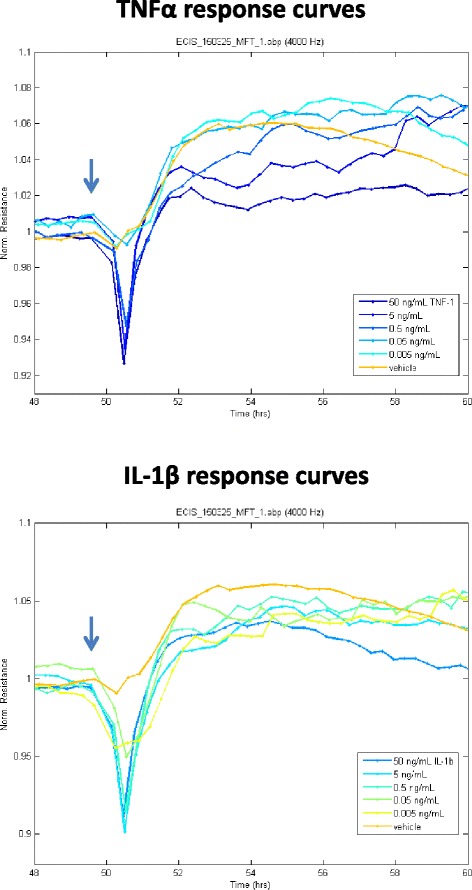
TNFα and IL-1β treatment causes acute reduction in the endothelial barrier resistance. ECIS-Ztheta technology was used to measure the barrier tightness, represented as barrier resistance. The *arrow* represents the time of cytokine addition, and the *colour-coded key* details the respective treatments. The TNFα responses are shown in the *upper graph* with the IL-1β responses shown in the *lower graph*. Each *curve* represents the mean of 4–6 wells

**Fig. 8:**
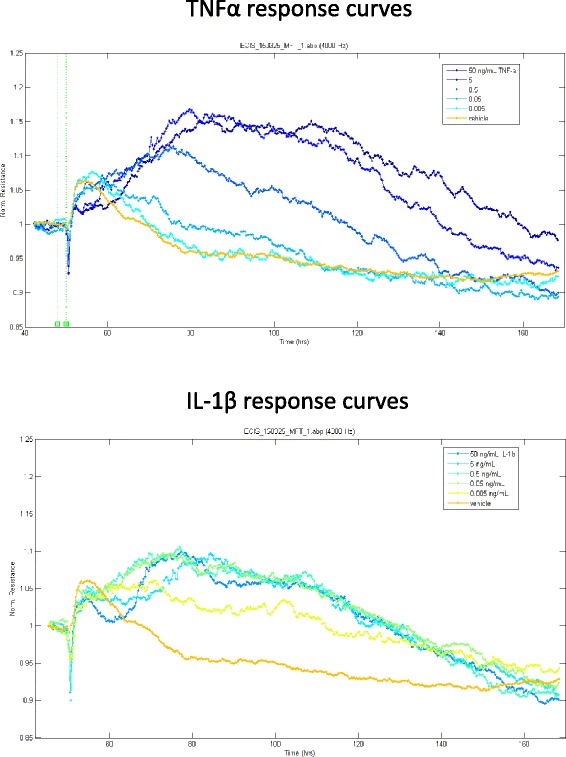
Following the acute reduction in barrier resistance, TNFα and IL-1β mediate a progressive and sustained strengthening of the endothelial barrier. The longer temporal profile measured by ECIS reveals that, following the initial small suppression of barrier resistance, there is a pronounced increase in barrier resistance from both IL-1β and TNFα, which occurs in a time- and concentration-dependent manner. Each curve represents the mean of 4–6 wells

Finally, we investigated the influence of these cytokines and inflammatory conditions on endothelial barrier function (Figs. 7 and 8 and Additional file 5: Figure S5). Barrier function was measured using the electric cell-substrate impedance-sensing (ECIS) ZΘ (Z-theta) instrument system from Applied Biophysics Inc (USA) [24] (see http://www.biophysics.com/barrierfunction.php). This system measures barrier function (as barrier resistance, ohms) in real-time and in a continuous autonomous manner. Each cytokine was tested from 5 pg/mL to 50 ng/mL, and endothelial TEER was monitored for more than 160 h. Basal TEER (normalised to 1.0) was relatively stable across this period and remained within 10 % of the TEER observed at the time of cytokine addition. Both TNFα and IL1β induced changes in TEER, but the effects were not identical. There was no net effect of TNFα at concentrations below 50 pg/mL, whereas at 500 pg/mL and above, there was a progressive and sustained increase in endothelial TEER. This increase in TEER was maximal at 5 ng/mL TNFα (Fig. 8 and Additional file 5: Figure S5).

The response to IL-1β was rather different, with the maximal increase in TEER observed with 500 pg/mL, and this relative increase in TEER was not as large as that seen with TNFα. Interestingly, at 5- and 50-ng/mL IL1β, there was a pronounced delay in the increase in TEER of approximately 20 h, which was not observed following TNFα treatment. These responses have been plotted against each other for direct comparison in Fig. 8 (see also Additional file 5: Figure S5). This reveals that the response profiles are quite different from each other, except at 500 pg/mL (Fig. 8).

## Discussion

To our knowledge this study represents the most comprehensive simultaneous analysis of cell-surface adhesion molecules and cytokine secretion following pro-inflammatory cytokine activation of human brain microvascular endothelial cells (hCMVECs). The goal was to ascertain the consequence of inflammatory activation on a broad range of immune mediators involved in BBB neuroinflammation, leukocyte communication and recruitment. The rationale was to advance our understanding of the repertoire of adhesion molecules, cytokines and chemokines expressed by brain endothelial cells and how these change in a temporal manner following activation by key inflammatory triggers (i.e. TNFα and IL-1β). Specific expression profiles for human brain endothelial cells is limited [27–30] (see Table 3), in comparison to endothelial cells derived from other tissues (e.g. HMEC-1 skin endothelial cells).

The brain endothelial cells used in this study express all of the prototypic adhesion molecules expected of endothelial cells; including CD31 (PECAM), CD34, CD44, CD144 (VE-cadherin) and the tight-junction-associated protein ZO-1. In addition, the hCMVECs have very low levels of CD62E, ICAM-1 and VCAM-1 under basal non-inflamed conditions. This is consistent with the phenotype of brain endothelial cells in vivo [31] and confirms that our in vitro culture conditions maintain the cells in a non-activated state. This is extremely important for being able to induce inflammation. As anticipated, both IL-1β and TNFα increased cell-surface expression of these key leukocyte-adhesion molecules; however, the potency of the response and temporal nature was different for induction of ICAM-1 and VCAM-1. Both were up-regulated to a greater extent and for longer by IL-1β. Elevated expression levels of CD62E, ICAM-1 and VCAM-1 are reliable indicators that endothelial cells have been activated in some manner. However, the temporal window of their expression is very specific. Most of the other adhesion molecules appeared unaffected by activation with IL-1β or TNFα. However, it is worthy to note that this conclusion is drawn from flow-cytometry analysis. As such, it is possible that compartmentalised localisation may change, which could then affect the function of the adhesion molecule.

Brain endothelial cells are the cellular interface between the blood and CNS tissue and like most cells with innate immune functions can release a myriad of cytokines in response to inflammatory challenge or damage. Here, we measured the release of 38 different factors, which were comprised of cytokines, chemokines, growth factors and soluble forms of receptors/adhesion molecules (see Table 1). In total, 13 of these were secreted, with most only after activation by IL-1β or TNFα. Interestingly, we observed the selective secretion of two CCL-class chemokines, MCP-1 (CCL2) and RANTES (CCL5), but no secretion of Eotaxin (CCL11), MIP-1α (CCL3) or MIP1β (CCL4). These are, however, sufficient to target a broad spectrum of immune-cell subsets expressing CCR1, CCR2, CCR3 and CCR5. Fractalkine (CX3CL1), the only known CX3CL chemokine, was not secreted by the endothelial cells under basal or activated conditions.

The CXCL chemokines (in our panel) released by the hCMVECs were IL-8 (CXCL-8) and IP-10 (CXCL10), whereas there was no secretion of either MIG (CXCL9) or I-TAC (CXCL11). IL-8 is a potent chemoattractant of neutrophils, which signals through the CXCR1 and CXCR2 receptors, IP-10 signals through CXCR3, which is expressed by subsets of T cells and monocytes/macrophages. Of particular importance to note was that the secretion of IP-10, GCSF and GMCSF, were almost exclusively induced by IL-1β, whereas the effects of TNFα were negligible in comparison. The increased GCSF and GMCSF maybe associated with creation of an environment within the CNS to support monocyte/macrophage differentiation post extravasation. The endothelial cells secrete an abundance of MCP-1 following activation, which is a potent chemokine for monocyte/macrophage subsets. In this study, TNFα increased the secretion of IL-8 and RANTES to a much greater extent than that induced by IL-1β. These differences suggest that the leukocyte recruitment signals released by the brain endothelial cells are considerably different between TNFα and IL1β responses.

Truncated versions of certain receptors and adhesion molecules can be liberated (cleaved or secreted) from the cell surface and consequently detected in the conditioned media. Here we measured the soluble forms of the IL-1 receptors, TNF receptors and ICAM-1 and VCAM-1. Soluble IL-1 receptors were not detected under any conditions; however, soluble TNFα receptors (both TNFRI and TNFRII) were detected. Interestingly, the amount was greater following IL-1β treatment, rather than TNFα treatment. The relevance of this novel finding is unclear but may relate to the desensitisation of the inflammatory environment to restrict TNFα signaling.

It is very interesting to note that TNFα appears to induce a much greater level of ICAM-1 and VCAM-1 on the surface of the endothelial cells, in comparison to IL-1β. This could be due to the fact that the level of soluble ICAM-1 and VCAM-1 present in the culture media was greater following IL-1β activation. This demonstrates that more ICAM-1 and VCAM-1 are cleaved during the IL-1β response (compare the data in Figs. 3 and 6). These adhesion molecules are the interacting partners of CD11 family members (e.g. LFA-1) and CD49d (VLA-4), respectively, which are broadly expressed by a range of leukocyte subsets. This data series nicely provides the basis to conduct adhesion/diapedesis experiments, which will be the subject of a future study. At this stage, it is exciting to hypothesise that TNFα and IL-1β differentially regulate tethering/attachment of different leukocyte subsets to the apical surface of the brain endothelium.

As well as being a comprehensive investigation of adhesion molecules important in the wider context of endothelial biology, we reveal expression of a number of adhesion molecules by hCMVEC not described previously for human brain endothelial cells. These include CD138, CD141, CD147, CD325 and CD50. This advances our understanding of their phenotype and adhesion-molecule expression profile, all of which are molecules important in endothelial functional biology.

A very important area clinically is the influence of neuroinflammatory events on vascular barrier function. Dysfunction or breakdown of the brain vascular integrity is a well-documented event in diseases such as relapsing remitting multiple sclerosis or in ischemic stroke patients, which can lead to vascular haemorrhage in some stroke patients. Surprisingly, there is a lack of studies comparing the effect of IL-1β and TNFα on barrier function using human brain endothelial cells (primary or cell line-derived) and advanced autonomous technologies such as ECIS. We therefore used ECIS-Ztheta system to monitor the effect of TNFα and IL1β on barrier function in a label-free temporal manner. Of specific importance for us was to determine whether there were any differences between the cytokine responses and to monitor the changes in barrier resistance either acutely (first few hours—24 h) or chronically over days. There was a small acute reduction in the endothelial barrier resistance in the first few hours with higher concentrations of TNFα (above 500 pg/mL) and IL-1β. This response was small, reflecting approximately 10–15 % difference in norm resistance. Reduced TEER over this period has been reported by others [32, 33]. After this acute period, the barrier resistance of the HCMVECs increased in a cytokine, concentration and time-dependant manner. This strengthening of the barrier occurs over a period, which is (surprisingly) omitted in other ECIS studies [32, 33]. The magnitude of the increase in barrier resistance is greater and occurs for longer for TNFα. This perhaps suggests that the brain endothelial cells are innately programmed to recognise elevated TNFα as a more dangerous signal than IL-1β and respond accordingly in terms of strengthening its integrity. This may also be the innate response to protect against any danger signals that may lead to neutrophil activation and invasion, which in terms of the brain vasculature, can be devastating. Our barrier resistance (ECIS) data for the hCMVECs highlight the importance of conducting acute and longer term analysis of barrier function, which we have achieved in a real-time autonomous manner using ECIS ZΘ. It is clear that this is an area which requires considerably more research to be conducted to understand the pathways leading to barrier protection.

This current study highlights the fact that these two potent inflammatory mediators induce different inflammatory responses from brain endothelial cells. This was originally suggested from the xCELLigence data, which measures the net changes in cellular focal adhesion. This therefore indicates that the brain endothelial cells are innately programmed to respond to different insults in specific ways. Therefore in vivo, certain diseases, infections or tissue injury may cause elevated levels of IL-1β, which may trigger different pathways and cytokine networks in the brain endothelium in comparison to conditions where TNFα is predominantly elevated (e.g. bacterial infection and autoimmune diseases).

### Future considerations

The data obtained in this study will be invaluable for investigating the dynamics of leukocyte attachment under different inflamed conditions and whether IL-1β induces recruitment of different subsets of immune cells in comparison to TNFα. The xCELLigence technology provides a cutting-edge tool to investigate the real-time temporal effects of activation of brain endothelial cells. In addition, the temporal sensitivity of ECIS for investigating cytokine changes in barrier function is incredibly powerful. This is clearly a complex area in the field of neuroinflammation, where there is still much to be discovered in terms of the endothelial responses and roles in disease. This will be applied to a range of activation signals and for conducting poly-pharmacological (multiple cytokines) analysis of how brain endothelial cells are regulated under more complex inflamed conditions.

## Additional files

Additional file 1: Figure S1. Gating strategy for the determination of live (P1) and dead gates (P2) for the cell-surface analysis of endothelial adhesion molecules. The live-cell exclusion dye 7AAD was used to determine the live-gate P1. 7AAD^+^ cells (detected in FL_3_) represent membrane-compromised or dead cells (P2). Typically, 7AAD^+^ cells were not present in P1. This gating strategy was conducted and applied in all flow-experiments. Additional file 2: Figure S2. Brain endothelial cytokine-secretion profile following treatment with the PKC agonist PMA. PMA suppressed basal production of VEGF, but the range of cytokines secreted following activation of PKC pathways was similar to that of IL-1β and TNFα. PMA (10 nM) treatment did not induce secretion of IL-1β and TNFα by the endothelial cells. Additional file 3: Figure S3. Flow-cytometry analysis of additional endothelial cell-surface molecules. The hCMVEC line expressed CD105, CD304 and CD141 basally, whereas basal expression of CD150 and DLL were negligible or undetected. None of these were substantially affected by IL-1β or TNFα treatment. Additional file 4: Figure S4. Expression of endothelial-specific tight-junction and adherens proteins. Immunocytochemical analysis of the distribution of VE-cadherin (CD144) and Zonula Occludin-1 (ZO-1) by the brain endothelial cells confirms the correct localisation of these two critically important endothelial-specific junctional proteins. The white arrows point to the location of the tight-junction complexes formed between two opposing endothelial cells. Nuclei and counter stained with Hoechst (blue). Additional file 5: Figure S5. Direct comparison of IL-1β and TNFα on endothelial barrier resistance measured by EICS-Ztheta. Comparisons are shown for 50 pg/mL, 500 pg/mL, 5 ng/mL, and 50 ng/mL. The colour coding is shown in the respective panel. In all cases, the control response is the orange curve. Data show the normalised resistance, which was measured at 4000 Hz for barrier function.
