# Supplementary material for: Pro-inflammatory TNFα and IL-1β differentially regulate the inflammatory phenotype of brain microvascular endothelial cells
Source: J Neuroinflammation. 2015 Jul 8;12:131. doi: 10.1186/s12974-015-0346-0 (PMC4506411; doi:10.1186/s12974-015-0346-0)
Supplement: Additional file 1: Figure S1. — Gating strategy for the determination of live (P1) and dead gates (P2) for the cell-surface analysis of endothelial adhesion molecules. The live-cell exclusion dye 7AAD was used to determine the live-gate P1. 7AAD+ cells (detected in FL3) represent membrane-compromised or dead cells (P2). Typically, 7AAD+ cells were not present in P1. This gating strategy was conducted and applied in all flow-experiments. [file 12974_2015_346_MOESM1_ESM.pptx]

## Slide 1
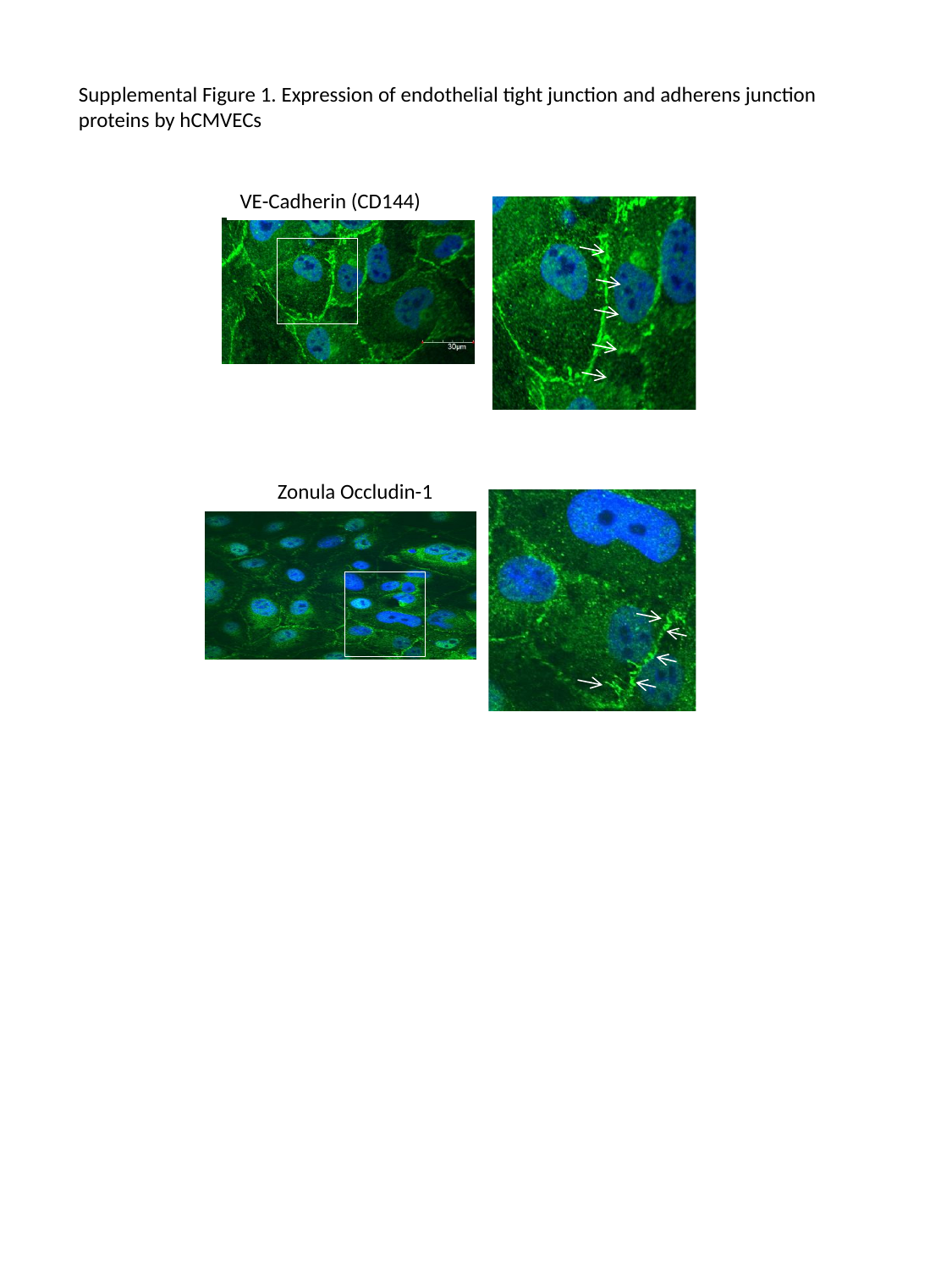

Supplemental Figure 1. Expression of endothelial tight junction and adherens junction proteins by hCMVECs
VE-Cadherin (CD144)
Zonula Occludin-1
