# Supplementary material for: Pro-inflammatory TNFα and IL-1β differentially regulate the inflammatory phenotype of brain microvascular endothelial cells
Source: J Neuroinflammation. 2015 Jul 8;12:131. doi: 10.1186/s12974-015-0346-0 (PMC4506411; doi:10.1186/s12974-015-0346-0)
Supplement: Additional file 2: Figure S2. — Brain endothelial cytokine-secretion profile following treatment with the PKC agonist PMA. PMA suppressed basal production of VEGF, but the range of cytokines secreted following activation of PKC pathways was similar to that of IL-1β and TNFα. PMA (10 nM) treatment did not induce secretion of IL-1β and TNFα by the endothelial cells. [file 12974_2015_346_MOESM2_ESM.pptx]

## Slide 1
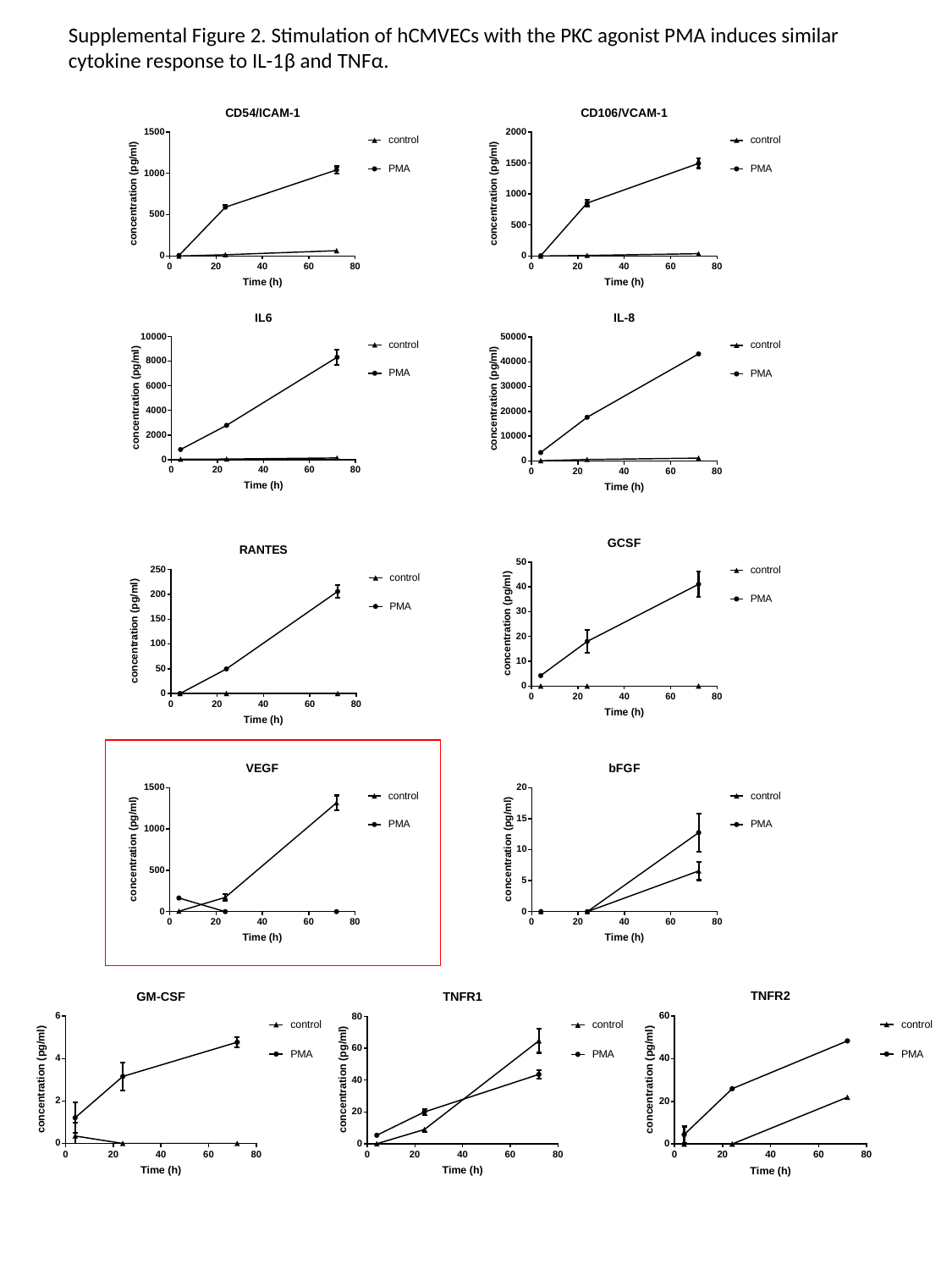

Supplemental Figure 2. Stimulation of hCMVECs with the PKC agonist PMA induces similar cytokine response to IL-1β and TNFα.
