# Supplementary material for: Pro-inflammatory TNFα and IL-1β differentially regulate the inflammatory phenotype of brain microvascular endothelial cells
Source: J Neuroinflammation. 2015 Jul 8;12:131. doi: 10.1186/s12974-015-0346-0 (PMC4506411; doi:10.1186/s12974-015-0346-0)
Supplement: Additional file 3: Figure S3. — Flow-cytometry analysis of additional endothelial cell-surface molecules. The hCMVEC line expressed CD105, CD304 and CD141 basally, whereas basal expression of CD150 and DLL were negligible or undetected. None of these were substantially affected by IL-1β or TNFα treatment. [file 12974_2015_346_MOESM3_ESM.pptx]

## Slide 1
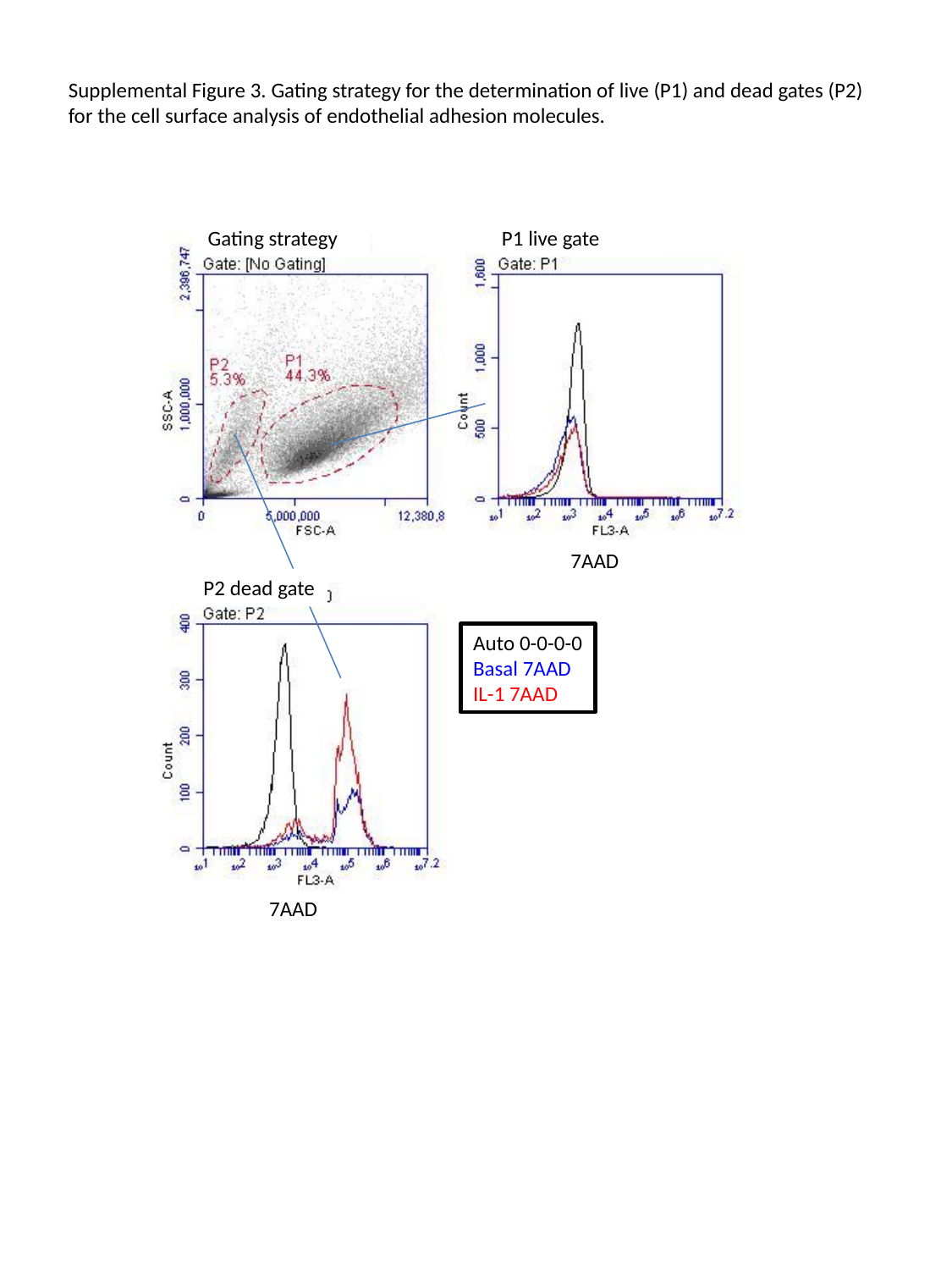

Supplemental Figure 3. Gating strategy for the determination of live (P1) and dead gates (P2) for the cell surface analysis of endothelial adhesion molecules.
Gating strategy
P1 live gate
7AAD
P2 dead gate
Auto 0-0-0-0
Basal 7AAD
IL-1 7AAD
7AAD
