# Supplementary material for: Pro-inflammatory TNFα and IL-1β differentially regulate the inflammatory phenotype of brain microvascular endothelial cells
Source: J Neuroinflammation. 2015 Jul 8;12:131. doi: 10.1186/s12974-015-0346-0 (PMC4506411; doi:10.1186/s12974-015-0346-0)
Supplement: Additional file 4: Figure S4. — Expression of endothelial-specific tight-junction and adherens proteins. Immunocytochemical analysis of the distribution of VE-cadherin (CD144) and Zonula Occludin-1 (ZO-1) by the brain endothelial cells confirms the correct localisation of these two critically important endothelial-specific junctional proteins. The white arrows point to the location of the tight-junction complexes formed between two opposing endothelial cells. Nuclei and counter stained with Hoechst (blue). [file 12974_2015_346_MOESM4_ESM.pptx]

## Slide 1
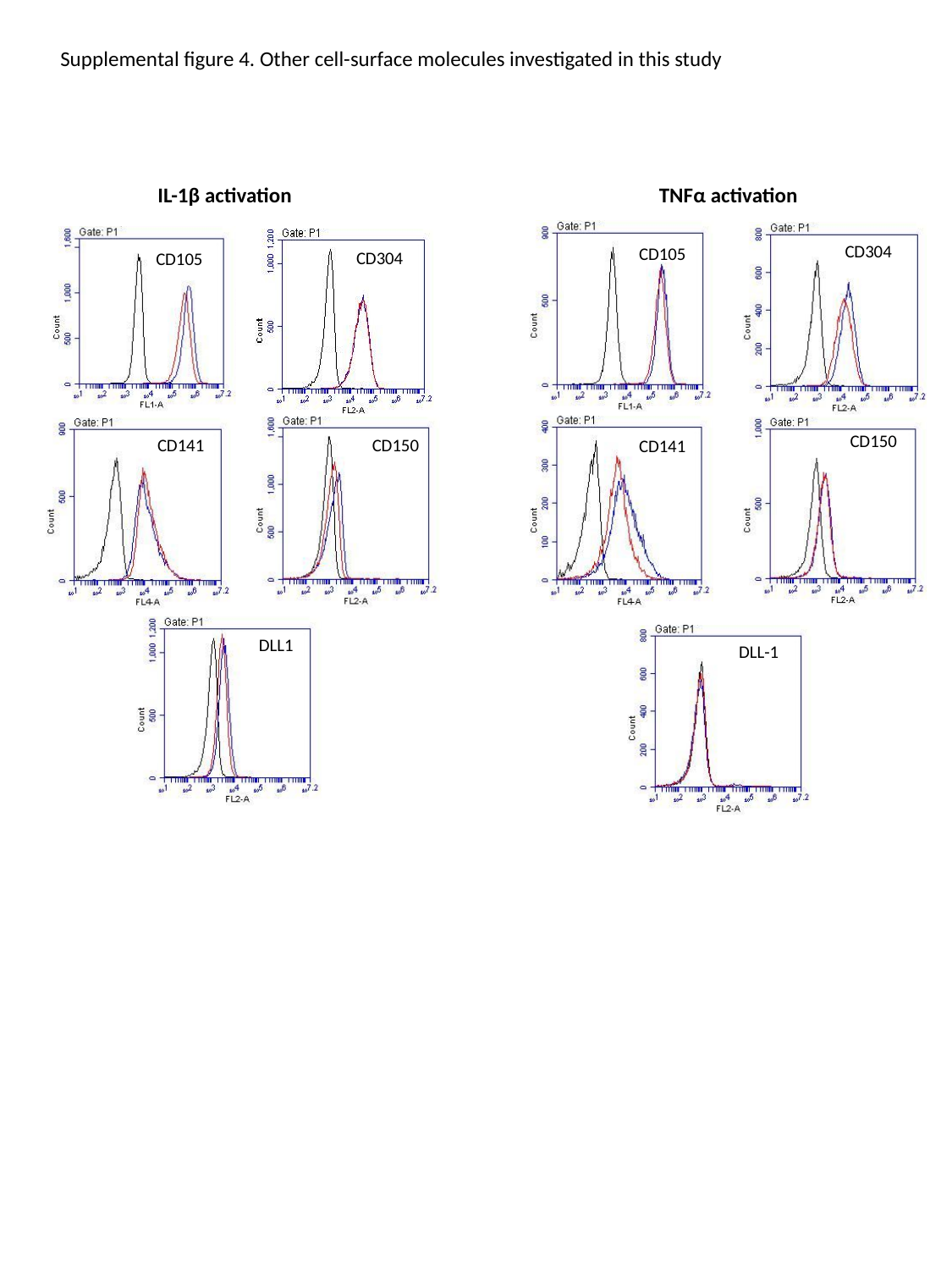

Supplemental figure 4. Other cell-surface molecules investigated in this study
IL-1β activation
TNFα activation
CD105
CD304
CD105
CD304
CD141
CD150
CD150
CD141
DLL1
DLL-1
