# Supplementary material for: Pro-inflammatory TNFα and IL-1β differentially regulate the inflammatory phenotype of brain microvascular endothelial cells
Source: J Neuroinflammation. 2015 Jul 8;12:131. doi: 10.1186/s12974-015-0346-0 (PMC4506411; doi:10.1186/s12974-015-0346-0)
Supplement: Additional file 5: Figure S5. — Direct comparison of IL-1β and TNFα on endothelial barrier resistance measured by EICS-Ztheta. Comparisons are shown for 50 pg/mL, 500 pg/mL, 5 ng/mL, and 50 ng/mL. The colour coding is shown in the respective panel. In all cases, the control response is the orange curve. Data show the normalised resistance, which was measured at 4000 Hz for barrier function. [file 12974_2015_346_MOESM5_ESM.pptx]

## Slide 1
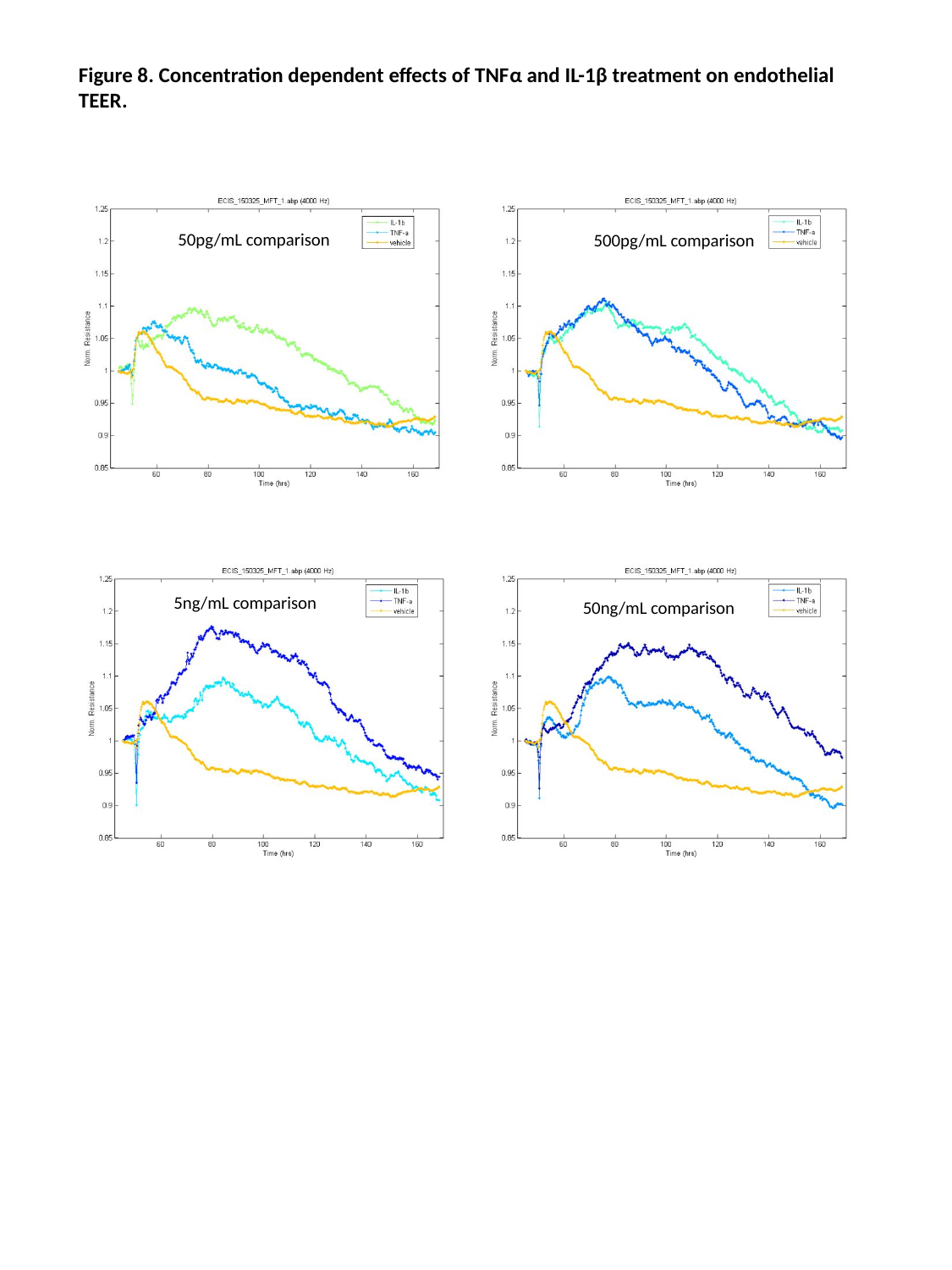

Figure 8. Concentration dependent effects of TNFα and IL-1β treatment on endothelial TEER.
50pg/mL comparison
500pg/mL comparison
5ng/mL comparison
50ng/mL comparison
